# Factors Associated with Periodontitis in Younger Individuals: A Scoping Review

**DOI:** 10.3390/jcm12206442

**Published:** 2023-10-10

**Authors:** Man Hung, Roah Kelly, Amir Mohajeri, Logan Reese, Sarah Badawi, Cole Frost, Taroniar Sevathas, Martin S. Lipsky

**Affiliations:** 1 College of Dental Medicine, Roseman University of Health Sciences, South Jordan, UT 84095, USA; 2 Department of Orthopaedic Surgery Operations, University of Utah, Salt Lake City, UT 84112, USA; 3 George E. Wahlen Department of Veterans Affairs Medical Center, Salt Lake City, UT 84148, USA; 4 Institute on Aging, Portland State University, Portland, OR 97201, USA

**Keywords:** dentistry, mouth diseases, periodontitis, public health

## Abstract

Periodontitis is a disease that affects many young adults, and if left untreated, it can have lasting and permanent effects on an individual’s oral health. The purpose of this scoping review was to review the recent literature to identify factors that place young individuals at risk of stage II or III periodontitis. Using the PRISMA guidelines for scoping reviews, three databases were systematically searched for peer-reviewed human studies published in English that investigated risk factors associated with stage II and/or III periodontitis in individuals less than 40 years of age. This review excluded abstracts, literature reviews, including narrative, scoping, and systematic reviews and meta-analyses, conference proceedings, letters to the editor, and editorials. The authors then extracted data from the relevant studies using a predefined form to summarize the aims, design, results, risk factors examined, and the type and severity of periodontitis. Among a total of 2676 articles screened, only three articles met the review’s inclusion criteria. Of these articles, one was a longitudinal case-control study and two were cross-sectional studies. Identified risk factors associated with stage II or III periodontitis included self-reported bleeding when brushing, low bone mineral density, being overweight, and smoking in young adults. Of note, only three studies met the inclusion criteria, suggesting a gap in the research literature.

## 1. Introduction

Periodontitis is a prevalent oral health condition [1,2] that impacts a significant portion of the adult population in the United States, affecting as many as two in five individuals. Initially, periodontitis begins as gingival inflammation, characterized by swelling and redness of the gum tissue caused by oral bacteria infecting tissue surrounding the tooth. These bacteria form a biofilm called plaque which hardens to become calculus [3].

The pathogenesis of periodontal disease is multifactorial, involving the interplay of bacterial biofilms, host response, and various predisposing factors [4,5,6,7]. The primary etiological factor is dental plaque, a biofilm containing a complex microbial community [8,9,10]. Specific bacterial species associated with periodontal pathogenesis, including *Porphyromonas gingivalis*, *Tannerella forsythia*, and *Treponema denticola*, are commonly referred to as the “red complex” bacteria [11]. These bacteria release virulence factors that directly destroy periodontal tissues and also trigger a host immune response. The host’s immune response to the microbial challenge is a key factor in periodontal disease progression [12,13]. An imbalance between pro-inflammatory cytokines, such as IL-1, IL-6, and TNF-α, and anti-inflammatory mediators leads to tissue damage. Additionally, neutrophils and other immune cells release reactive oxygen species and enzymes, like matrix metalloproteinases, adding to the cycle of connective tissue breakdown and bone resorption [14]. Factors that can modify the susceptibility to periodontal diseases include genetics, smoking, systemic diseases (e.g., diabetes), medications, stress, and hormonal changes [5,15,16].

Periodontal disease in younger individuals can differ from that in older adults. Gingivitis is the most common form in children and adolescents [17,18]. Often linked to poor oral hygiene, gingivitis does not damage the tooth’s supporting structures and is usually reversible with appropriate oral care. Aggressive periodontitis (also known as juvenile periodontitis) affects younger individuals and is characterized by a rapid loss of periodontal support. It often shows a familial predilection, suggesting a genetic predisposition. Aggregatibacter actinomycetemcomitans has been strongly associated with aggressive periodontitis [19]. Some systemic conditions that affect children, such as Papillon–Lefèvre syndrome or neutropenia, can predispose young individuals to severe periodontal destruction [5,20]. The aggressive nature of periodontal diseases among younger individuals merits early and aggressive management.

As periodontitis advances, it can instigate a range of oral health issues, notably tooth migration and drifting. This means that affected teeth may shift from their original positions, leading to misalignment and irregular spacing in the dental arch. Consequently, individuals with periodontitis often experience masticatory dysfunction, or difficulties with chewing and biting, due to these structural alterations within their mouths [21]. As untreated periodontitis deteriorates, it weakens the foundation that supports the teeth, eventually resulting in tooth mobility and, in some cases, complete tooth loss [22]. This not only has a negative impact on an individual’s quality of life but also directly affects aspects related to function and esthetics [23].

In 2017, the World Workshop [24] on the Classification of Periodontal and Peri-Implant Diseases and Conditions introduced a classification system that categorized periodontal diseases into four distinct stages. Stage II is defined as 3 to 4 mm of interdental clinical attachment loss, with 15 to 33% radiographic bone loss [25]. Stage III is defined as interdental clinical attachment loss greater than or equal to 5 mm, radiographic bone loss extending to the middle third of the root and beyond, and loss of four or fewer teeth. This review focused on these stages because of the risk and danger that come with the disease’s progression, from irreversible bone loss to impaired dentition. 

Beyond its direct effects on oral health, periodontitis has been linked to an array of chronic systemic diseases, such as cardiovascular disease, diabetes, endocrine diseases, musculoskeletal conditions, reproductive system abnormalities, and respiratory infections [26]. The inflammation and bacterial pathogens associated with periodontitis can potentially exacerbate these systemic health issues or increase the risk of developing them, making periodontitis a critical concern for overall well-being.

Numerous studies have shed light on the prevalence of periodontal disease, revealing a concerning trend that underscores its significance in the context of aging. These investigations have consistently shown that the likelihood of developing periodontitis escalates with advancing age [27], with a staggering statistic emerging: by the time individuals reach the age of 65 and beyond, an astonishing 70% of them will exhibit some degree of gum disease [28]. These data underscore the pressing need for targeted interventions and vigilance in identifying patients who are at risk for periodontitis, not only to prevent its onset but also for early detection and timely intervention.

While the link between age and periodontal disease is well documented, it is imperative to recognize that this condition’s impact extends far beyond the confines of oral health alone. The association between periodontitis and systemic health concerns has sharpened the focus on identifying susceptible individuals, as addressing this issue can have far-reaching health implications [29]. Thus, the imperative to identify at-risk patients is twofold: to prevent the progression of periodontal disease and to potentially mitigate its systemic repercussions.

While several studies have investigated risk factors and behaviors associated with periodontal disease, most of these studies focused on older adults. Fewer studies examine risk factors for moderate to severe periodontitis in younger individuals. Although more frequent in older adults, periodontal disease in younger individuals may be a distinct clinical entity that can be rapidly progressive, more severe, and exhibit a variable response to treatment [30]. Its aggressive nature and adverse clinical and psychological impact make it important to understand who is at greatest risk [31]. Moreover, the incidence of early-onset aggressive periodontal disease among younger individuals seems to be on the rise, further underscoring the urgency of identifying those who are most at risk [32,33]. As this trend gains momentum, it becomes increasingly vital to comprehend the unique risk factors associated with this subgroup of patients, as doing so can not only improve our understanding of the disease but also pave the way for more targeted prevention and treatment strategies.

In summary, periodontal disease exhibits a clear age-related trend, with a substantial proportion of elderly individuals experiencing its effects [34]. However, the implications of this disease transcend age, affecting both oral and systemic health. Recognizing individuals at risk for periodontitis is paramount, not only for older adults but also for the younger population, where early-onset aggressive forms of the disease are emerging. By identifying and addressing these risk factors, we can curtail the impact of periodontitis on individuals’ health and well-being across their lifespan.

The goal of this scoping review was to provide an overview of the recent research examining risk factors for periodontitis among younger adults. In addition to summarizing the current literature, this review sought to map the breadth and depth of evidence on the topic and identify potential gaps in knowledge; thus, it lends itself to a scoping review approach [35]. This review should assist dentists and others interested in oral health to develop preventative strategies and early interventions to target those at greatest risk to help improve the oral health of young adults.

## 2. Methods

This scoping review adhered to the rigorous guidelines set forth by PRISMA (Preferred Reporting Items for Systematic Reviews and Meta-Analyses) for scoping reviews [36], ensuring a methodical and standardized approach to the assessment of the available literature. The primary objective of this review was to evaluate and synthesize peer-reviewed human studies published during the period from 2013 to 2023. Specifically, the focus of this review was on research that delved into the identification and examination of risk factors associated with stage II and/or stage III periodontitis in individuals aged 40 years or younger.

To ensure that the review captured the most up-to-date insights into the field of periodontitis, the inclusion criteria were tailored to encompass only those articles published within the past decade, spanning from 2013 to 2023. This focus was instrumental in summarizing the latest advancements in our understanding of the risk factors associated with stage II and stage III periodontitis among younger individuals. By limiting the scope to this specific timeframe, the review aimed to provide current synthesis of knowledge that could inform future research directions and clinical practices aimed at the prevention and management of these severe stages of periodontal disease.

The exclusion criteria consisted of published abstracts without the full text, review articles, conference proceedings, opinion pieces, and letters to the editor. This review also focused on modifiable factors and excluded studies related to genetic syndromes and anatomic abnormalities. Table 1 summarizes the review’s inclusion and exclusion criteria. 

The authors searched 3 databases: PubMed, Web of Science (WOS), and Cochrane. Table 2 outlines the specific search strategies and keywords for each database. 

Five authors (R.K, C.F., S.B., L.R., and T.S.) independently screened the titles and abstracts of the articles generated by the database searches to assess whether an article potentially met the inclusion criteria. After completing the screening process, the five authors met to review their selections and to decide which articles should be included or excluded. The full text of potentially relevant articles was then reviewed by all five authors to confirm if a study met the review criteria. For relevant articles, the authors generated and used a predefined data extraction form to summarize information about article type, study design, participants’ characteristics, and outcomes. To achieve consensus, the authors collectively reviewed the data extraction form to identify and resolve differences. Another author (MH) audited the quality, sources of evidence, and data charting to confirm the validity of the results.

## 3. Results

### 3.1. Study Selection

**Figure 1:**
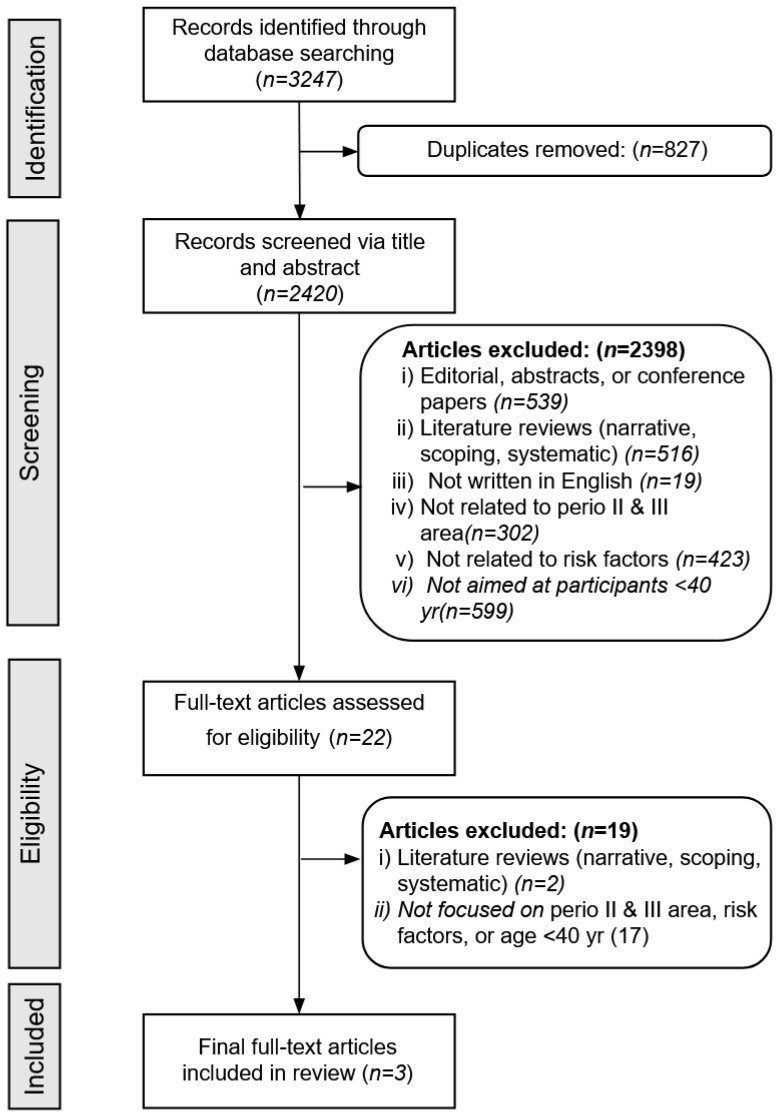
Flowchart of the article selection process.

Using the criteria outlined in Table 1, the initial searches of the PubMed, WOS, and Cochrane databases yielded a total of 3247 articles. After removing duplicates, 2420 articles remained for screening. Another 2398 articles were excluded because they were either editorials, abstracts, or conference papers (539), literature reviews (516), not written in English (19), not related to periodontitis stages II and III (302), did not study risk factors (423), or did not study participants <40 years old (599). The remaining 22 studies were assessed in detail, resulting in 3 articles that met all the review criteria. Figure 1 provides an overview of the selection process.

### 3.2. Study Features

Table 3 presents a summary of the studies. Of the three studies included in this review, two were conducted in Brazil and one in China. The sample size ranged from 29 to 2032 participants, and all of the study participants fell within the age range from 18 to 39 years old. The research designs included two cross-sectional studies and one longitudinal study.

### 3.3. Findings

The two most common risk factors for periodontitis were “smoking” and “being overweight” [37]. Other risk identified factors were “gums bleeding” and “low bone density” [37,38]. Compared to non-smokers, smoking tobacco caused greater gingival recession and gingival pocket depths and a decrease in tissue repair and immune response [39].

Two articles found an association between patients’ weight and gingival health [37,38]. These both found that overweight or obese non-pregnant women and men were at a greater risk of severe periodontal disease than normal-weight individuals. Both studies found a correlation between eating habits and overall health [37,38], which directly affected the health of the gingival tissue. In contrast, Jiang et al. [38] did not find an association between smoking and periodontitis. While they found the direction of overweight/obese women as being more likely to have periodontal disease, their results were not strong enough to draw firm conclusions.

Finally, Costa et al. explored the correlation between bone density and periodontal disease [11]. They found that patients with lower bone density have an increased risk of periodontitis stages II and III [37]. Conversely, individuals with higher bone density appear to be at a lower risk.

**Table 3:** Study summaries.

Author (Year)	Country	Sample Size	Age Range (Years)	Study Aims	Study Design	Outcomes
Da Silva et al., 2022 [39]	Brazil	2022	26–40	Investigated the influence of smoking on clinical, microbiological, and immunological parameters in young adults with stage III-IV, Grade C periodontitis	Longitudinal, case-control study	Young smokers with stage III-IV grade disease responded less favorably to treatment for all study parameters at both 3 and 6 months
Jiang et al., 2016 [38]	China	987	18–40	Examined the prevalence and risk factors of periodontal disease among Chinese women preconception	Cross-sectional	Women with bleeding during brushing are at increased risk of periodontal disease. Smoking and obesity were not significant factors
Costa et al., 2021 [37]	Brazil	2032	18–19	Investigated the association between low bone mineral density (BMD) and severe periodontitis	Cross-sectional design	Low BMD is associated with both the severity and extent of periodontitis in adolescents. Secondary results found education and income inversely related to severe disease

## 4. Discussion

While the modifiable risk factors for periodontitis have been extensively studied and acknowledged, there remains a notable gap in our understanding of the specific risk factors associated with this condition among younger adults. Since periodontal disease in younger adults is specifically classified and because it may differ from disease in older adults, it could have distinct risk factors. A key finding is that our review yielded only three articles published since 2013 that addressed this age group, identifying a gap in the available evidence and highlighting the need for additional study.

Among the studies, smoking and being overweight or obese were identified as significant risk factors associated with periodontitis in younger adults. Da Silva et al. [39] examined the influence of smoking on periodontal health and inflammatory markers in patients who received full-mouth ultrasonic debridement coupled with the use of amoxicillin and metronidazole. Their findings revealed a significant association between smoking and higher probing depth, bleeding on probing, and gingival recession compared to non-smokers after treatment. This aligns with previous research that identified smoking as a risk factor that negatively impacts periodontal health [40]. For example, Tomar and Asma found that smokers experienced a significantly higher prevalence of periodontitis than non-smokers and that about half of periodontitis cases among US adults could be attributed to cigarette smoking [41]. Delving deeper into the mechanisms at play, it becomes evident that the deleterious effects of smoking on periodontitis are multifaceted [42,43,44]. Plaque bacteria, recognized as pivotal players in the pathogenesis of periodontitis, act as the catalysts for a poorly controlled inflammatory response that can wreak havoc on periodontal tissues [45,46,47]. A cascade of events sets the stage for a slew of adverse consequences, including periodontal tissue damage, the formation of pathological pockets around the teeth, and ultimately, the unsettling loosening and loss of teeth [48]. De Silva et al. [39] found fewer pathogenic bacterial species, *Porphyromonas gingivalis and Fusobacterium nucleatum,* in non-smokers, suggesting a potential mechanism for how smoking exacerbates periodontitis. It implies that smoking may create a more hospitable environment for these pathogenic bacteria to flourish, perpetuating the destructive cycle of periodontal disease.

Furthermore, obesity is another risk factor for periodontitis. Obesity is not solely a cosmetic issue but also has significant implications for a person’s overall health. The link between obesity and periodontitis can be traced to the role of adipose tissue in inflammation. While once thought of as metabolically inactive, adipose tissue is an active endocrine organ that secretes a variety of substances, including inflammatory cytokines [49,50]. These cytokines, such as tumor necrosis factor-alpha (TNF-α) and interleukin-6 (IL-6), play pivotal roles in the body’s inflammatory responses [51,52,53]. An excessive release of these cytokines from the expanded adipose tissue seen in obese individuals leads to a heightened inflammatory state, which increases susceptibility to various inflammatory diseases, including periodontitis [54,55]. Suvan et al. examined the relationship between obesity and periodontal health and found a significant association between obesity and periodontitis. Overweight individuals were found to have both a higher prevalence of periodontitis and more severe disease compared to their normal-weight counterparts [56]. This suggests that excess adipose tissue contributes to the inflammatory processes that exacerbate periodontal disease. In a separate study, Chaffee and Weston also found a significant correlation between obesity and the likelihood of developing periodontal disease. Specifically, they found that obese individuals face a 35% higher risk of developing periodontitis compared to non-obese individuals [57]. This underscores the connection between maintaining a healthy weight and good periodontal health. Costa et al. [37] investigated the association between low bone mineral density and periodontitis and found that individuals with reduced lumbar bone mineral density were more likely to exhibit severe periodontitis. This suggests a possible opportunity for early intervention to slow disease progression by promoting healthy bone forming behaviors, such as adequate calcium and vitamin D intake, and weight bearing exercise. In a parallel line of inquiry, Costa et al. [37] also noted an association between overweight/obesity and severe periodontitis. Young adults are gaining weight faster than any age group, and obesity-related comorbidities such as high blood pressure and type 2 diabetes often begin in this age group [58]. This review suggests that periodontitis merits inclusion in the list of chronic health conditions triggered at an early age by being overweight or obese.

Jiang et al. [38] likewise identified a trend between overweight individuals and a higher risk of periodontitis, but they could not draw firm conclusions due to the lack of statistical significance [38]. However, the observed trend aligns with Costa et al. and other studies that identify being overweight as a potential risk factor for periodontitis [59]. Additionally, Jiang et al. did not find an association with several other potential risk factors such as smoking, alcohol use, and socioeconomic factors. These results differ from De Silva et al. and Costa et al. and also underscore the need for more in-depth study of risk factors for early-onset periodontitis. 

### 4.1. Limitations

It is important to acknowledge and address the limitations inherent in this review as they provide valuable context for interpreting the findings and considering the broader implications of this study. A primary limitation worth noting is the relatively constrained pool of studies that met the stringent inclusion criteria set forth for this review. The retrieval of only three relevant studies, while reflective of the rigorous selection process, does place certain constraints on the breadth and generalizability of the conclusions drawn from this analysis. One avenue for potentially mitigating this limitation could have been to broaden the search criteria and expand the exploration across a wider array of databases. However, this approach carries its own set of caveats and potential drawbacks. Expanding the search criteria might indeed yield a larger quantity of studies, but it could also inadvertently introduce a larger volume of studies with varying levels of quality and relevance. This could potentially dilute the overall robustness and reliability of the findings, undermining the integrity of the review. Thus, the decision to maintain strict inclusion criteria was made to prioritize the quality and relevance of the studies included, ensuring that the findings would be based on a solid foundation of research.

Additionally, another limitation to consider is the geographical scope of the studies included in this review. As only two countries are represented in the selected studies, there is a potential limitation regarding the generalizability of the findings to broader, more diverse populations. Variations in cultural practices, healthcare access, socioeconomic factors, and genetic predispositions among different populations can influence the prevalence and impact of risk factors for periodontitis. Therefore, caution should be exercised when extrapolating the results to populations beyond the specific regions and demographics covered in these studies.

### 4.2. Implications

The insights gleaned from this scoping review have significance for healthcare practitioners, including physicians, dentists, and other health professionals with opportunities to provide their patients with optimal care and guidance. Understanding risk factors identifies who might benefit from more assertive preventative interventions and early treatment. Such tailored guidance and proactive dental care can play a pivotal role in thwarting the progression of periodontal disease, particularly in the younger population.

Furthermore, while risk factors contributing to periodontitis have been extensively explored, it is worth noting that this review found only three relevant studies specifically pertaining to younger individuals. This finding underscores the pressing need for additional research in this area. By delving deeper into the distinct risk factors that exert the most significant influence on the younger demographic, healthcare professionals can refine their approach to prevention and treatment. This deeper understanding can inform the development of personalized and nuanced strategies tailored to each patient’s unique profile, thereby mitigating the burden of early-onset periodontitis.

By recognizing the risk factors and their differential impact across age groups, healthcare providers can take a proactive stance in their practice. They can incorporate these insights into their patient care protocols, implement specific prevention strategies, and craft individualized treatment plans that address the specific needs of younger individuals at risk of developing periodontitis. The ultimate goal is to reduce the prevalence and severity of periodontitis among younger adults, promoting both their oral health and overall well-being.

Additionally, the findings of this scoping review have practical implications for both healthcare providers and the general public. Healthcare providers, dentists, and primary care providers can be more proactive in screening for periodontitis, especially when they encounter young adults with one or more of these risk factors. The interconnectedness between oral health (periodontitis) and overall health (smoking, weight, and bone density) emphasizes the need for a holistic approach to patient care. Providers might consider inter-professional collaboration, where a patient with periodontitis could be referred for a bone density scan or nutritional counseling. For individuals, understanding these associations can empower young adults to seek regular dental check-ups, especially if they identify with one or more risk factors. An awareness of risk factors and the importance of oral health might also lead to better oral hygiene practices and healthier lifestyle choices, potentially preventing or delaying the onset of periodontitis. Governments and public health organizations might consider incorporating these findings when designing public health campaigns. For instance, campaigns that target smoking cessation might also describe the risk of periodontitis among other health risks. Similarly, campaigns promoting healthy weight and diet could incorporate messages about the importance of oral health.

### 4.3. Conclusions

This scoping review found that smoking, being overweight, having gums that bleed when brushing, and low bone density are associated with periodontitis in young adults. Although this topic needs additional study, these findings provide a start to understanding the risk factors for periodontitis in young adults and to developing strategies for prevention and intervention to address them. Healthcare professionals and policymakers can chart a course toward more effective periodontal health promotion, fostering not only healthier smiles but also improved overall well-being among young adults.

## Figures and Tables

**Table 1:** Inclusion and exclusion criteria.

Inclusion Criteria	Exclusion Criteria
Peer-reviewed in English Participants aged <40 years Focused on risk factors for stage II and/or III periodontitis Article published between 2013 and 2023 Subjects must be human	Articles that consisted of only abstracts without the full text Animal studies Literature reviews (narrative, scoping, systematic, meta-analysis) Conference Proceedings Letters for editors Studies related to genetic syndromes and anatomic abnormalities

**Table 2:** Search strategies.

Databases	Search Strategies	Number of Articles Found
PubMed	((“risk factors”[Title/Abstract] OR “causal”[Title/Abstract]) AND (“periodontal disease”[Title/Abstract] OR “periodontitis”[Title/Abstract] OR “Aggressive Periodontitis”[Title/Abstract] OR “Periapical Periodontitis”[Title/Abstract] OR “Chronic Periodontitis”[Title/Abstract] OR “Early-Onset periodontitis”[Title/Abstract] OR “Juvenile Periodontitis”[Title/Abstract])) AND ((humans[Filter]) AND (2013:2023[pdat]))	1326
Web of Science	(TS = (“risk factors” OR causal)) AND TS = (“periodontal disease” OR periodontitis OR “Aggressive Periodontitis” OR “Periapical Periodontitis” OR “Chronic Periodontitis” OR “Early-Onset periodontitis” OR “Juvenile Periodontitis”) AND ((ALL = ((“population groups” not “animal models”))) OR ALL = (men OR women OR patient OR female OR male OR subjects OR adult)) NOT ALL = (“animal models”)	1716
Cochrane	#1 (“risk factors” OR causal):ti,ab,kw AND (“periodontal disease” OR periodontitis OR “Aggressive Periodontitis” OR “Periapical Periodontitis” OR “Chronic Periodontitis” OR “Early-Onset periodontitis” OR “Juvenile Periodontitis”):ti,ab,kw (Word variations have been searched) #2 (men OR women OR patient OR female OR male OR subjects OR adult NOT animal):ti,ab,kw #3 #1 AND #2	282

## Data Availability

Not applicable.
